# Interleukin-1 Blockade in Polygenic Autoinflammatory Disorders: Where Are We now?

**DOI:** 10.3389/fphar.2020.619273

**Published:** 2021-01-26

**Authors:** Hana Malcova, Tomas Milota, Zuzana Strizova, Dita Cebecauerova, Ilja Striz, Anna Sediva, Rudolf Horvath

**Affiliations:** ^1^ Department of Paediatric and Adult Rheumatology, University Hospital Motol, Prague, Czechia; ^2^ Department of Immunology, Second Faculty of Medicine, Charles University and University Hospital Motol, Prague, Czechia; ^3^ Department of Clinical Immunology and Allergology, Institute for Clinical and Experimental Medicine, Prague, Czech Republic

**Keywords:** systemic juvenile idiopathic arthritis, adult-onset Still´s disease, idiopathic recurrent pericarditis, IL-1, anakinra, canakinumab, rilonacept

## Abstract

Polygenic autoinflammatory diseases (AIDs), such as systemic juvenile idiopathic arthritis (sJIA), adult-onset Still's disease, Kawasaki disease, idiopathic recurrent pericarditis (IRP), Behçet’s Syndrome, Crystal-induced arthropatihes such as gout or Calcium pyrophosphate deposition disease are characterized by the overexpression of inflammasome-associated genes, leading to a dysregulation of the innate immune response. The IL-1 cytokine family (IL-1α, IL-1β, IL-1Ra, IL-18, IL-36Ra, IL-36α, IL-37, IL-36β, IL-36g, IL-38, IL-33) was defined to be principally responsible for the inflammatory nature of polygenic AIDs. Several clinical trials were initiated, and IL-1 blockade has been proven to cause a rapid reduction of clinical symptoms and normalization of laboratory parameters in the majority of cases. Randomized, placebo-controlled, clinical trials, together with registry-based clinical trials and open-label, retrospective and prospective observational studies, supported the efficacy and safety of IL-1 inhibitors in the treatment of polygenic AIDs. Most of the current data are focused on the therapeutic use of anakinra, an IL-1 receptor antagonist, canakinumab, an anti-IL-1β monoclonal antibody, and rilonacept, a soluble decoy receptor. However, other promising agents, such as gevokizumab, IL-1β blocking monoclonal antibody, tadekinig alfa, a human recombinant IL-18-binding protein, and tranilast, an analog of a tryptophan metabolite, are currently being tested. Anakinra, canakinumab and rilonacept caused impressive improvements in both systemic and musculoskeletal symptoms. Furthermore, the anti-IL-1 therapy allowed corticosteroid tapering and, in some cases, even withdrawal. This article reviews the current IL-1 inhibitors and the results of all clinical trials in which they have been tested for the management of broad spectrum of polygenic AIDs.

## Introduction

Polygenic autoinflammatory diseases (AIDs) are designated as a category of complex multifactorial diseases of unknown etiology characterized by a dysregulation of innate immune responses and the overexpression of inflammasome-associated genes. Although polygenic AIDs share clinical features with monogenic AIDs, multiple factors are involved in disease pathogenesis (Krainer et al., 2020). The polygenic AIDs typically consist of systemic juvenile idiopathic arthritis (SJIA), adult-onset Still's disease (AOSD) and Behçet’s Syndrome (BD) (Rigante 2018); however, other disease entities, such as Kawasaki disease (KD) and idiopathic recurrent pericarditis (IRP) disease, gout or Calcium pyrophosphate deposition disease (CPDD) are also related (Cantarini et al., 2015; Patel and Shulman 2015). Symptomatology overlap with monogenic AIDs, including recurrent fevers, musculoskeletal symptoms, and serositis, is well known. Nevertheless, life-threatening complications, such as macrophage activation syndrome (MAS) and secondary amyloidosis, may also appear in polygenic AIDs (Shenoi 2017; Giacomelli et al., 2018). Moreover, the polygenic AIDs share common pathophysiological features such as hyperactivation of inflammasome and the overproduction of the IL-1 cytokine family. The role of Interleukin (IL) -1 cytokines is also discussed in many other seemingly unrelated conditions such as atherosclerosis, heart failure, cardiomyopathy or Type 2 diabetes mellitus (Cavalli and Dinarello 2018; Szekely and Arbel 2018; Gram 2020).

**FIGURE 1:**
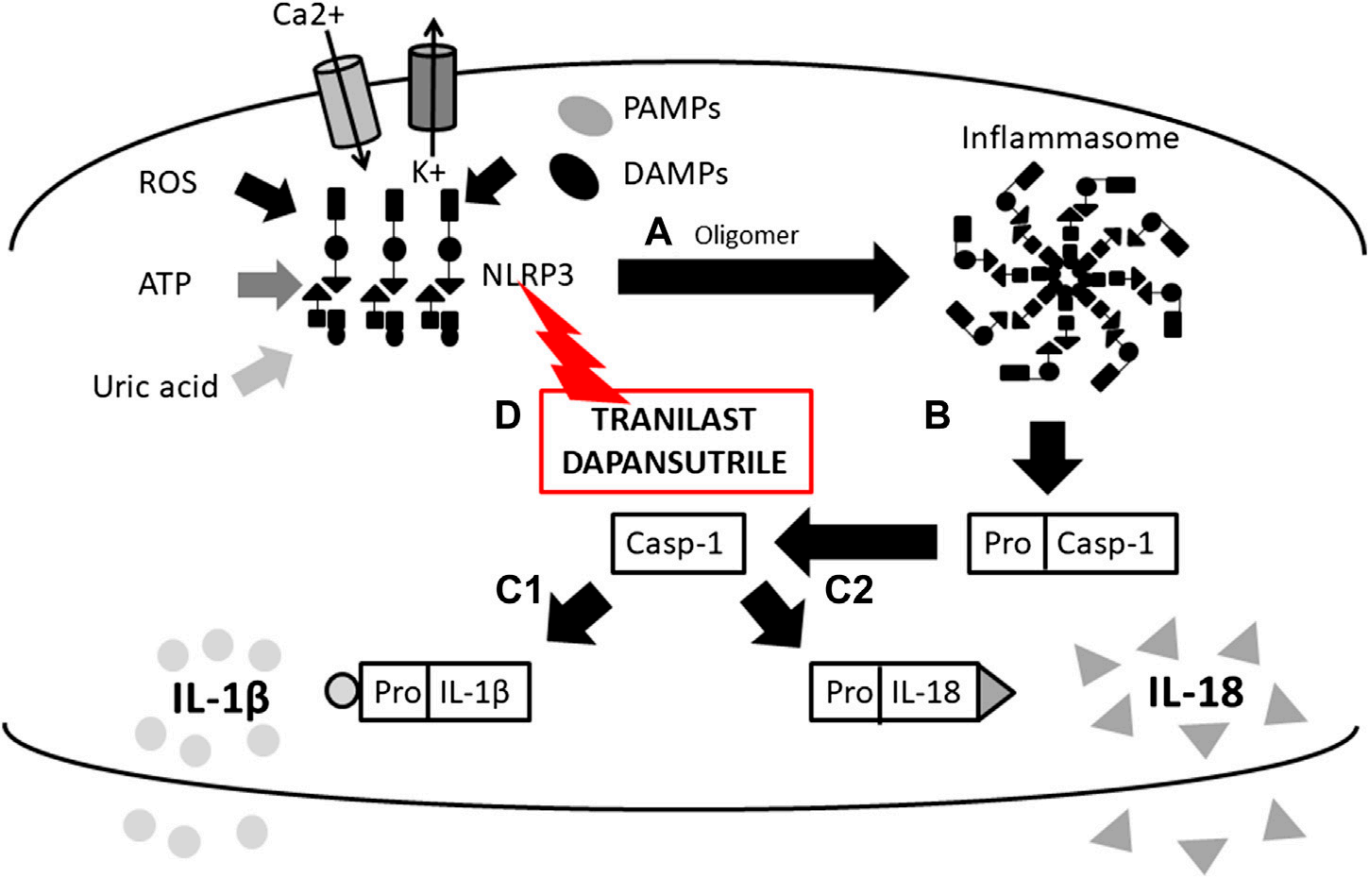
*Scheme of IL-1β and IL-18 activation mediated by inflammasome and mechanims of inhibition*. (A) initiation of NLRP3 oligomerization (by DAMPs, PAMPs, ROS, UA, potassium eflux, calcium influx), (B) cleavage of pro-caspase 1 N-terminal region (inactive form) by the inlfammasome molecular complex, (c-1) IL-1β and (c-2) IL-18 activation from pro- IL-1β and pro- IL-18 inactive form by caspase-1 and release from the cell, (D) inflammasome inhibition with ***Tranilast*** (direct inhibitor of NLRP3) (NLRP3, Nucleotide-binding oligomerization domain, Leucine rich Repeat and Pyrin domain containing 3; DAMPs, Damage-Associated Molecular Patterns; PAMPs, Pathogen-Associated Molecular Patterns; ROS, Reactive Oxygen Species; UA, Uric Acid).

**FIGURE 2:**
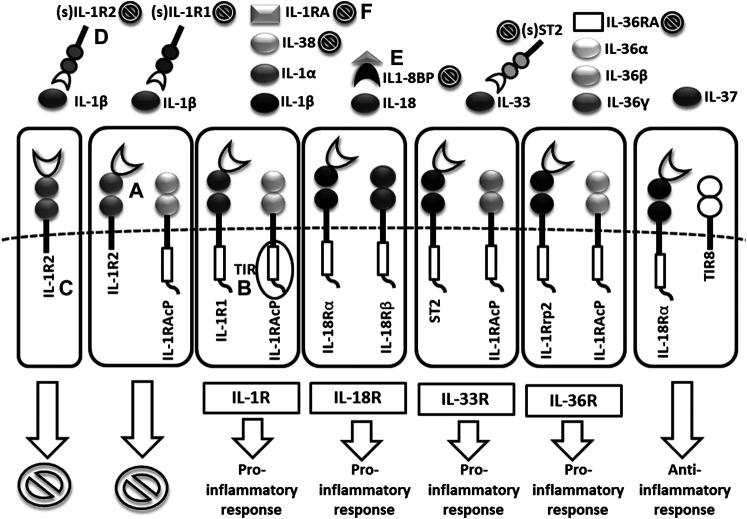
*Scheme of IL1 receptor family structures and mechanisms of regulation*. (A) cell membrane receptors structure of binary complexes–primary (IL-1R1, IL-18Rα, ST2, IL-1Rrp2) and accessory receptors (IL1-RAcP, IL-18Rβ), (B) signal transmission via TIR domains, (C) regulatory role of TIR-less receptors (IL1-R2) binding cytokines without signal transmission (inhibition ), (D) regulatory role of soluble receptors (IL1-R1, IL1-R2, ST2) and (E) binding proteins (IL18-BP) binding cytokines without signal transmission, (F) inhibitory role of receptor antagonists (IL-1Ra, IL-36Ra, IL-38) (TIR = Toll/interleukin-1 receptor).

Cytokines in the IL-1 family are molecules that play a crucial role in the immune system functioning. To date, 11 structurally and functionally diverse cytokines in the IL-1 family have been described. Most of the cytokines in the IL-1 family are produced as inactive precursors. These precursors are further activated intracellularly by molecular cleavage. The process is mediated by Caspases 1, 3, 7 (Yuan and Akey 2013; Kelley et al., 2019; Yang et al., 2019) and other proteases such as calpain, elastase or chymase produced by innate immune cells (Clancy et al., 2018). All of them are of major importance for the activation of the IL-1 cytokine family and, therefore, for the initiation of the inflammatory immune response. The caspases can be triggered by different stimuli leading to either an inflammatory immune response (caspase 1) or apoptosis (caspase 3 and 7). The active forms of cytokines (IL-1β, IL-18, IL-36) are then released from a cell (Figure 1). In a different scenario, the activated IL-1α and IL-33 may be also stored intracellularly and released as alarmins from damaged cells (Dinarello, 2018; Scott et al., 2018). IL-1α may in some cases also serve as a membrane-bound cytokine that is constitutively expressed on epithelial and endothelial cells and therefore contributes to local inflammatory reactions. (Di Paolo and Shayakhmetov 2016). The active cytokine forms bind to a specific receptor and serves either as receptor agonists (IL-1α, IL-1β, IL-18, IL-33, IL-36α, IL-36β, IL-36γ) or antagonists (IL-1Ra, IL-36Ra, IL-38) (Boraschi et al., 2018) and generate pro-inflammatory (IL-1α, IL-1β, IL-18, IL-33, IL-36α, IL-36β, IL-36γ) or anti-inflammatory immune responses (IL-1RA, IL-36RA, IL-37, IL-38) (Figure 2) (Fields et al., 2019).

**FIGURE 3:**
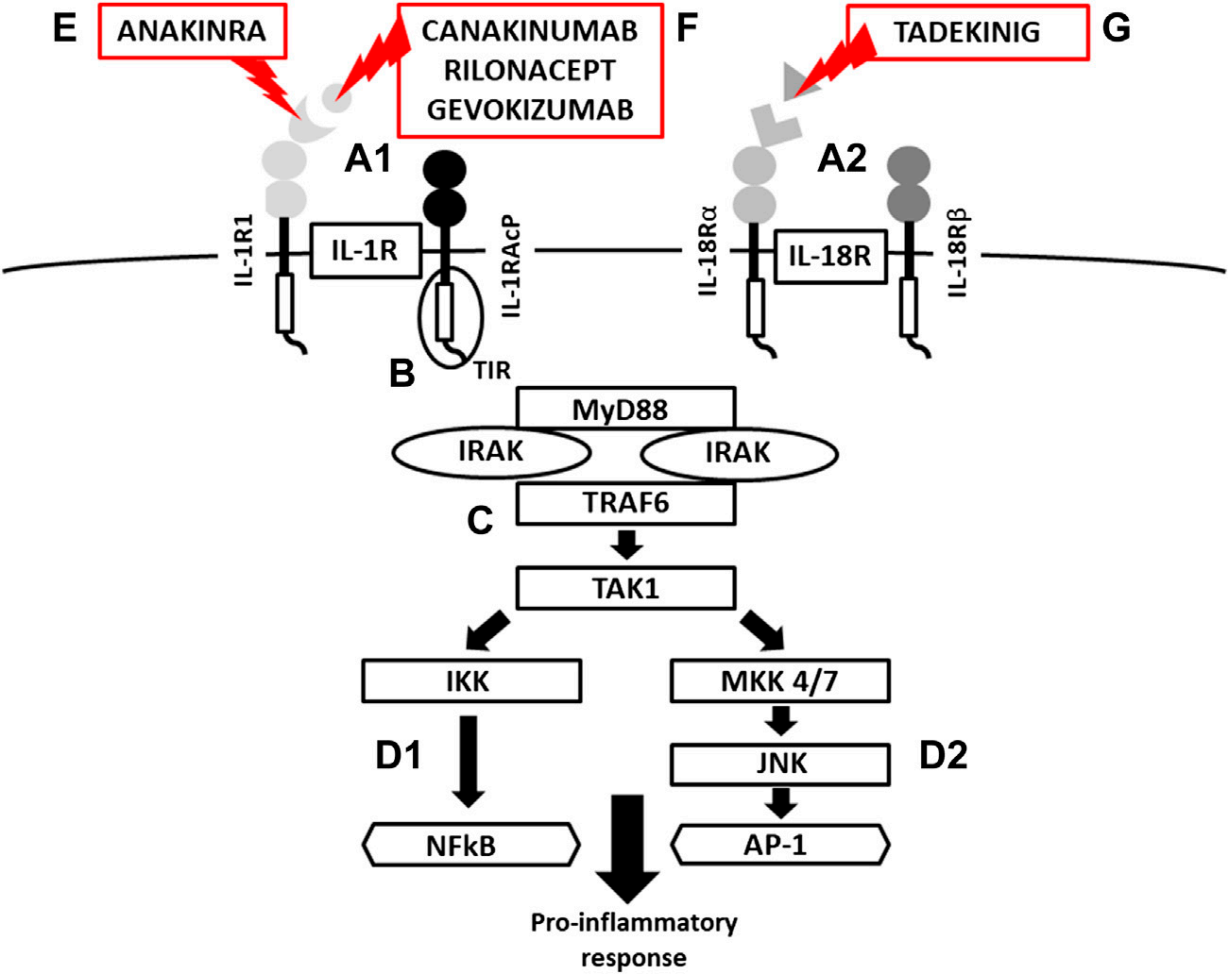
*Scheme of IL-1R and IL-18 activation, signaling and mechanism of inhibition*. (A) IL-1/IL-18 binding cell membrane IL-1R/IL-18 receptors (structure of binary complexes), (B) signal transmission via TIR domains, (C) initiation of pro-inflammatory response via IRAK-MyD88-TRAF6-TAK1 pathway, activation of transcription factors NFkB via IKK and (d-1) AP-1 via JNK-MKK4/7 (MAP kinases) (d-2), (E) IL-1R blockade by **Anakinra** (a recombinant antagonist of the IL-1 receptor **, (F)** IL-1β inhibition by **canakinumab** and **gevokizumab** (monoclonal antibodies neutralizing IL-1β), **Rilonacept** (a soluble receptor predominantly binding IL-1β), (G) IL-18 inhibition by **Tadekinig alfa** (a human recombinant IL-18 binding protein) (TIR = Toll/Interleukin-1 Receptor domain**,** MyD88 = Myeloid Differentiation primary response 88, IRAK = Interleukin-1 Receptor Associated kinase, TRAF6 = TNF Receptor Associated Factor 6, TAK1 = Transforming growth factor beta-Activated kinase 1, IKK = NFkB Inhibitor kinase, NFkB = Nuclear Factor Kappa B, MAP = Mitogen Activated Protein kinase, JNK = c-Jun N-terminal kinase AP1 = Activator Protein 1).

**FIGURE 4:**
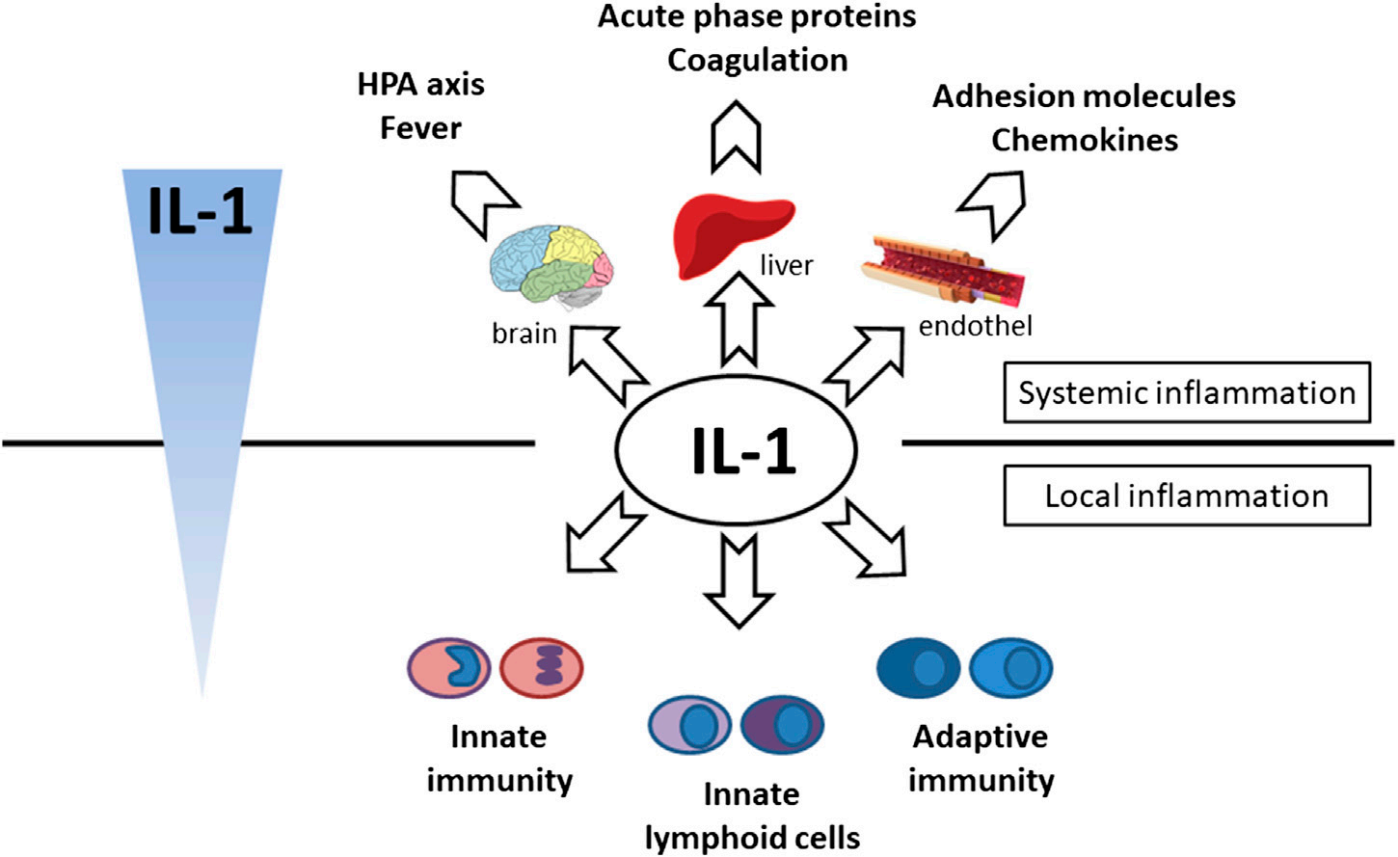
*Effector function of IL-1 cytokine*. **Systemic effect**—hypothalamic–pituitary–adrenal axis activation (cortisol production and fever induction), production of acute phase proteins (CRP, SAA, ferritin, complement) and coagulation factors (fibrinogen) production in liver, endothel activation (expression of chemokines and adhesion molecules), **local effect**—activation of innate immunity (monocytes, neutrophils), adaptive immunity (Th17, CD8) and innate lymphoid cells (ILC3, γδT17).

Following the binding of pro-inflammatory cytokines in the IL-1 family downstream signaling cascade leads to activation of crucial transcription factor Nuclear factor kappa B (NFkB) initiating the production of pro-inflammatory cytokines (IL-1, IL-2, IL-6, IL-8, IL-12, etc.) and chemokines (CCL2, CCL5, CXCL2, CXCL1, CXCL8, CXCL10). NFkB, however, is also associated with the expression of cell cycle regulators (cyclins), pro-apoptotic proteins (Bcl-2, IAPs), and adhesion molecules (ICAM-1, VCAM-1) (Liu et al., 2017). Together with NFkB, the transcription factor AP-1 also functions as a significant regulator of cell proliferation, differentiation, and transformation (Figure 3) (Hess et al., 2004; Zhang et al., 2017). On the other hand, several cytokines, such as IL-1a and IL-33, have their own transcriptional activity and are thus capable of entering the cell nucleus and binding to DNA (Werman et al., 2004). Since the overactivation of inflammatory immune responses through inflammatory cytokines may lead, in some cases, to a massive and possibly life-threatening systemic reaction, each step in the inflammatory response is regulated by multiple mechanisms. These regulatory mechanisms consist of membrane receptors, which lack the intracellular TIR domain and inhibit the cytokine-receptor interaction (IL1-R2), receptor antagonists (IL-1RA, IL-36RA, IL-38), soluble receptors (IL1-R2, ST2, receptor complexes ST2/IL-1RAcP or IL1-R2/IL-1RAcP binding free IL-1α and IL-1β, IL-33) or binding proteins (IL-18BP). Among other inhibitory molecules involved in the inflammatory immune responses, an atypical receptor TIR8, serves as a negative regulator. IL-37 also has a direct anti-inflammatory effect by controlling the expression of transforming growth factor β (TGFβ), the anti-inflammatory cytokine. The IL-1 cytokines and IL-1 family related receptors are expressed in different kinds of tissues and in very variable amount, thus overproduction of IL-1 cytokines may lead to both systemic as well as local inflammation (Figure 4) (Garlanda et al., 2013; Mantovani et al., 2019). The identification of strong IL-1 involvement in the pathogenesis of polygenic AIDs has revealed a great potential of IL-1 inhibitors in the treatment of these uncommon disorders (Feist et al., 10-2018). Moreover, the significant therapeutic effect of IL-1 inhibitors in the treatment of AIDs highlighted the importance of IL-1 cytokines in the disease pathogenesis (Dinarello, 2011; Dinarello, 2019). Anakinra, a recombinant form of the IL-1 receptor antagonist (IL1-RA) (accessed on March 8, 2019), canakinumab, a human monoclonal antibody blocking the interaction between IL-1β and the IL-1 receptor (European Medicines Agency, 2019b), and rilonacept, a soluble receptor predominantly blocking IL-1β (Tarp, et al., 2016; Junge et al., 2017), are the most prevalent anti-IL-1 therapies in polygenic AIDs. Other promising agents include gevokizumab, an IL-1β neutralizing monoclonal antibody (Owyang et al., 2011), tadekinig alfa, a human recombinant IL-18 binding protein (Gabay et al., 2018), tranilast, an analog of a tryptophan metabolite (Huang et al., 2018) or dapansutrile, a direct selective inhibitor of NLRP3 inflammasome (Kluck et al., 4-2020) (Figures 1 and 2). In this review, we have collected all data regarding anti-IL-1 therapies in polygenic AID patients. We have provided a brief overview of the selected polygenic AIDs and further focused on randomized, placebo-controlled, clinical trials, together with registry-based clinical trials, and open-label, retrospective and prospective observational studies (Tables 1A–F). The aim was to call attention to the treatment possibilities in such challenging disorders, such as polygenic AIDs.

**TABLE 1:** A list of observational, open-label, randomized placebo-controlled, and registry-based clinical trials with IL-1 inhibitors in the treatment of **(A)** SJIA, **(B)** AOSD, **(C)** IRP, **(D)** KD.

Study population	Treatment	Study design	Time of follow-up	Outcomes	Author (year)
A. Systemic juvenile idiopathic arthritis					
9 pts. (7 F, 2 M; age range 4–17 yrs)	Anakinra (2 mg/kg, max 100 mg s.c./day)	Prospective open label	6.6 months	●reduction of systemic and musculoskeletal symptoms	Pascual et al. (2005)
				●GC sparring effect	
				●mild ISRs in all patients	
22 pts. (11 F, 11 M; age range 0.9–18.7 yrs)	Anakinra (1 mg/kg, max 100 mg s.c./day)	Prospective open-label	1.36 years	● rapid response to therapy with reduction of systemic and musculoskeletal symptoms	Gattorno et al. (2008)
				● possible discontinuation of all other treatment	
				● fewer affected joints and higher absolute neutrophil count as the response predictors	
86 (initial phase)/50 (blinded phase)/44 pts. (extension)	Anakinra (1 mg/kg, max 100 mg s.c./day) or placebo	Randomized blinded placebo-controlled study with an open-label run-in period followed by an open-label extension	12 wks - 12 months	●ISRs, RTI, headaches, arthralgias, fever and abdominal pain as the most common AEs	Ilowite et al. (2009)
				●favourable reponse to therapy- progressive improvement in CHAQ and reduction of ESR	
				●systemic features, fewer disease flares and shorter time to flare as response predictors	
24 pts. (15 F, 9 M; mean age 8. yrs.)	Anakinra (2 mg/kg, max 100 mg s.c./day) or placebo	Randomised double-blind placebo-controlled trial followed by open label extension	1–12 months	●favourable response to the therapy	Quartier et al. (2011)
				●mild ISRs as the most common ISR	
46 pts. (27 F, 19 M; median age 7.6 yrs)	Anakinra	Retrospective	12.5 months	●rapid relief of systemic features, musculoskeletal symptoms less responsive	Nigrovic et al. (2011)
				●early treatment initiation as prevention of persistent synovitis	
				●normalization of inflammatory markers within 1 month	
				●younger age at the onset and higher as response predictors	
24 pts. (16 F, 8 M; mean age 12.6 yrs)	Rilonacept (up to 4.4 mg/kg or 320 mg s.c./wk.) or placebo	Randomized double blind placebo-controlled study followed by open label phase	4 wks – 12 months	●no significant difference in response between the placebo and the rilonacept group at week 4	Lovell et al. (2013)
				●favourable response rates of ACR pedi 30/50/70 at month 12	
				●MRP8/MRP14 and D-dimer evaluated as the new potential biomarkers	
				●mild ISRs as the most common AEs, no SAEs reported	
71 pts. (46 F, 25 M; median age 9.5–10.5 yrs)	Rilonacept (initial dose 4.4 mg/kg up to 320 mg s.c., maintenance 2.2 mg/kg up to max 160 mg s.c.once/wk.) or placebo	Randomized double blind placebo-controlled study followed by open label phase	4 wks – 21 months	●shorter time to response and higher response rate in rilonacept	Ilowite et al. (2014)
				● no influence on response of previous exposition to anakinra and absence of systemic manifestation	
				●significant GC-sparing effect	
				●SAEs associated with the relapse of the underlying disease	
20 pts. (13 M, 7 M; mean age 7.9 yrs)	Anakinra (2 mg/kg, max 100 mg s.c./day)	Prospective open-label	32 months	●favourable response at month 1- prompt body temperature normalization and decrease of inflammatory markers	Vastert et al. (2014)
				●IL-18 and/or S100A12 or S100A8/9 as potential biomarkers	
				●no SAEs, ISRs as the most common AEs	
25 pts. (13 M, 12 F; mean age 7.3 yrs)	Anakinra	Retrospective	2.8 years	●persisting clinically inactive disease	Pardeo et al. (2015)
				●possible withdrawal of GC and DMARDs	
				●shorter time from disease onset to treatment initiation as response predictors	
				●ISRs as the most common AEs	
77 pts. (40 F, 37 M; median age 3.8 yrs)	Anakinra, canakinumab, tocilizumab, etanarcept, adalimumab, abatacept	Retrospective registry-based	Up to 33.8 months	●higher response rate and longer interval of persistence on treatment in pts. with IL-1/il-6 inhibitors compared to TNFα	Woerner et al. (2015)
				●lower drug survival of anakinra as second/third-line compared to canakinumab or tocilizumab therapy after month 12	
				● GC sparring effect	
				●no differences in incidence of AEs	
30 pts. (25 F, 5 M; median age 5.7 yrs)	Anakinra (initial therapy), potential switch to canakinumab	Registry-based	39.9 weeks	●clinically inactive disease	Kimura et al. (2017)
				●persistent response after GC tapering	
216 children (age range 2–12 yrs), 56 young adolescents (12–16 yrs), and 29 older adolescents or young adults (>16 yrs)	Canakinumab	Pooled analysis	12 weeks	●prompt response at day 15, persisted response at day 85	Feist et al. (2018) (Feist, Quartier et al., 2018)
				●AEs observed in 86.7–88.3% of pts	
177 children (98 F, 79 M; median age 8 yrs At the time of study initiation)	Canakinumab 4 mg/kg up to 300 mg s.c./4 weeks	Open-label extension of pivotal trials	271 weeks	●LDA (JADAS) in 48.6% pts.; aJIA-ACR response 50/70/90 in 73.4%/65.5%/52%	Ruperto et al. (2018)
				●GCs discontinuation in 51/128 (39.8%) pts	
				●better response in biologic naive pts	
				●SAEs incidence 40.68/100 pt-yrs; infection rate 10.28/100 pt-yrs	
77 pts. (43F, 34 M, mean age 12.7 yrs)	Anakinra, canakinumab	Retrospective	Up to 60 months	●comparable drug retention on anakinra and canakinumab therapy	Sota et al. (2018) (Sota, Insalaco et al., 2018)
				●higher retention rate in pts. with shorter disease duration and biologic-naive	
				●no impact of csDMARD comedication, GC-sparing effect	
				●ISRs as the most common AEs	
76 pts. (43 F, 33 M; mean age 7 yrs)	Anakinra, tocilizumab	Registry-based	12 months	●MDA, ACR pedi 90 and CID in 51%, 42% and 39% pts. at year 1	Kearsley-Fleet et al. (2019)
				●differences between tocilizumab and anakinra not significant	
				●higher treatment survival rate observed in tocilizumab	
Part I:166 (90 F, 76M; mean age 9 yrs)/Part II: (39 F, 36 M; mean age 10.5–11 yrs)	Part I: canakinumab 4 mg/kg s.c./4 wks/Part II: dose 2–1-0 mg/kg s.c. (arm I); interval 8–12–0 wks (Arm II)	Two-part open-label	24 wks (Part II duration)	●dose reduction or interval prolongation possible in sJIA pts. without flare	Quarteir et al. (2020)
				●treatment discontinuation possible in limited number of pts	
123 pts. (75 F, 48M; mean age 4.3–6.5 yrs, 8.2–10.5 yrs)	Canakinumab 4 mg/kg s.c./4 weeks	Long-term open-label	2 yrs (Study duration)	●similar response to therapy between fever and non-fever pts	Brunner et al. (2020)
				●use of MTX and no GC at baseline as response predictors	
				●GC-free status higher in patients with early response	
				●higher infection rate in fever group and in patients previously exposed to GC and bDMARDs	
				●ACR pedi 50 as the strongest predictor of achieving JADAS clinical remission	

SJIA, Systemic Juvenile Idiopathic Arthritis; AOSD, Adult-Onset Still's dDisease; IRP, Idiopathic Recurrent Pericarditis; KD, Kawasaki Disease; F, Female; M, Male; yr/s., year/s; mo., month/s; wk/s., week/s; pt/s, patient/s; s.c., subcutaneous; ESR, Erythrocyte Sedimentation Rate; CRP, C-Reactive Protein; S/AEs, Severe/Adverse Event/s; ISRs, Injection Site Reaction/s; ACR (Pedi), (Pediatric) American College of Rheumatology response; LDA, Low Disease Activity; JADAS, Juvenile Arthritis Disease Activity Score; BDCAF, Bechçet's dDisease Current Activity Form; BSAS, Bechçet's Syndrome Activity Score; CHAQ, Childhood Health Assessment Questionnaire; BDQOL, Bechçet's dDisease Quality of Life; PGA, Physician Global Assessment; PGE, Patient Global Evaluation; DMARDs, Disease-Modifying Antirheumatic Drugs; TNFα, Tumor Necrosis Factor alpha; GCs, Glucocorticosteroids.

## Methods

We conducted a comprehensive review of the literature on the efficacy and safety of IL-1 inhibition therapy. Anakinra, rilonacept and canakinumab, in SJIA, AOSD, IRP, KD, BS, gout, CPPD and appropriate combinations were used as the key words in the search strategy. Only English written and peer-reviewed reports published in indexed international journals until October 2020 were reviewed. The databases used for the search included Medline/PubMed and Web of Science. The authors followed proposed guidelines for biomedical narrative review preparation (Gasparyan et al., 2011).

### Systemic Juvenile Idiopathic Arthritis

SJIA is a distinct subtype of juvenile idiopathic arthritis. This multifactorial autoinflammatory disorder with a typical manifestation in the childhood exhibits a wide range of clinical phenotypes ranging from a systemic course of the disease with a high spiking fever and a skin rash to a chronic and destructive arthritis (Cimaz, 2016; Lee and Schneider, 2018). Other complications of SJIA include Macrophage Activation Syndrome (MAS) that belongs to one the most redoubtable and life-threatening manifestation of SJIA with high mortality. MAS is characterized by typical clinical course and laboratory findings including particularly fever, splenomegaly, hepatopathy, coagulopathic and pancytopenia. Chronic inflammation may lead to secondary amyloidosis, which also contributes to mortality and morbidity of SJIA patients (Shenoi and Wallace, 2016). Until recently, SJIA therapy was based primarily on the long-term administration of high-dose systemic glucocorticosteroids (GCs) and was associated with numerous side effects and devastating consequences, especially in pediatric patients. However, there is now evidence that IL-1 and IL-6 play crucial roles in the pathogenesis of SJIA. Therefore, biologic therapy, such as IL-1 inhibition, has become one of the leading therapeutic strategies to control the disease (Cimaz. 2016). Several clinical and observational studies have already evaluated the efficacy of the IL-1 inhibitors anakinra, rilonacept and canakinumab in patients with SJIA.

### Interleukin-1 Blockade in the Treatment of SJIA

The essential role of IL-1 inhibitors was proposed by Pascual et al. (2005), who demonstrated the upregulated gene expression of the IL-1 cytokine in patients with SJIA. Based on these findings, therapy with anakinra, a potent IL-1R antagonist, was provided to 9 children with active disease who were refractory to other treatments. Seven of them achieved complete remission, and the other two patients had a partial response with significantly improved clinical symptoms and laboratory parameters. The improvement occurred in both systemic (relief of fever) as well as musculoskeletal symptoms (complete resolution of arthritis score) within first week. Interestingly, serum cytokine levels did not correlate with disease activity (Pascual et al., 2005). Similar results were obtained by Gattorno et al. (2008). However, two different patterns of response were seen within the first week of therapy. In the first group, patients reported an immediate improvement in systemic and joint manifestations as well as a decrease in inflammatory markers. In the second group, despite of initial improvement, that was seen at baseline; the patients tended to relapse subsequently in terms of disease activity, especially joint involvement. Patients in the first group who reported a complete response to treatment had considerably fewer joints with active disease and higher absolute neutrophil counts. Similar to the study by Pascual et al. “*in vitro*” secretion (spontaneous or LPS induced) of IL-1β and IL-18 did not correlate with the disease activity and was not affected by the treatment (Gattorno et al., 2008). These findings were in contrast to the prospective study by Vastert et al. (2014). The authors demonstrated an excellent response in almost all patients in whom anakinra was used as the first-line therapy. A prompt response was seen in body temperature normalization, which occurred in 80% of patients within 3 days upon treatment initiation. Afterward, decreases in inflammatory markers (CRP, ESR, ferritin) and achievement of an adapted ACR Pedi 90 response occurred in 80% of patients within 30 days of treatment, which persisted for up to 3 years. However, approximately one-third of the patients required concomitant medication to maintain remission. The study further identified two potential biomarkers, I-L18 and/or S100A12 or S100A8/9, which might serve as tools for the management of the cessation of recombinant IL-1Ra treatment in patients with SJIA (Vastert et al., 2014).

Nevertheless, the results of previous studies with anakinra were limited due to the open-label study design. One of the first randomized double-blind placebo-controlled clinical trials by Ilowite et al. (2009) was primarily focused on the treatment safety. Treatment-related complications consisted mainly of injection site reactions (ISRs) which were retreating in time and the authors, thus, showed favourable safety profile of anakinra. The most of AEs were reported as mild or moderate and only single AE was consider as severe (SAE). Moreover, the preliminary results showed that more than a half of the patients responded to the therapy (improvement >30% in JRA Core Set Criteria). More responders were seen in the patients with systemic manifestations (73% vs. 67% with musculoskeletal symptoms). Moreover, fewer disease flares, shorter time to flare, and progressive improvements in CHAQ and ESR were observed in anakinra-treated patients (Ilowite et al., 2009). Promising results of the previous study were later confirmed by Quartier et al. (2011) in ANAJIS trial**.** After one month of treatment, a complete response (ACR Pedi 30, absence of fever, reduction of ESR and/or CRP) as primary endpoint was achieved in 67% of the patients treated with anakinra and only in a single patient receiving a placebo. High efficacy rate was also achieved in patients upon switch from placebo to anakinra—90% of them responded within two months. Interestingly, the overexpression of type I IFN-inducible genes was observed after initiation of anakinra treatment regardless of the clinical response (Quartier et al., 2011).

However, retrospective and registry-based studies reflecting a “real world” evidence also provided valuable information. In a large retrospective study by Nigrovic et al. (2011), almost 60% of the patients (including 8 of 10 children) receiving anakinra monotherapy as the first-line treatment attained a complete response. Almost all patients displayed rapid improvement in fever, rash and return of the inflammatory parameters to normal within the first month, while arthritis was less responsive to the treatment. It has been also suggested that the early initiation of anakinra as the concept of “window of opportunity” may prevent the development of persistent synovitis. Patients with a partial or no response differed from patients with a complete response with regard to a younger age at the onset of the disease (mean age 5.2 years vs. 10.2 years; *p* = 0.004). There was also a significant difference in ferritin levels, which were higher in patients with a complete response than in patients with a partial response (1329 ng/ml vs. 3008 ng/ml) (Nigrovic et al., 2011). These observations indicate that patients with greater monocytemacrophage system activation respond better to IL-1 inhibition. This phenomenon has also been noted in other studies (Lequerre et al., 2008; Nigrovic et al., 2011; Hedrich et al., 2012; Romano et al., 2014; Pardeo et al., 2015; Grom et al., 2016; Horneff et al., 2017; Kearsley-Fleet et al., 2019; Vastert et al., 2019). Comparable results were obtained in a retrospective study by Pardeo et al. (2015). (Pardeo et al., 2015). Another important aspect of the successful treatment is represented by the Drug Retention Rate (DRR). Retrospective study by Sota et al. (2018) attempted to identify the factors influencing the DRR level of anakinra or canakinumab. Cumulative DDR on these drugs ranged from 79.9% at month 12–53.5% at month 48 and remained unchanged until month 60. No differences were found between anakinra and canakinumab and between patients treated with monotherapy or with a combination therapy with csDMARDs (conventional synthetic disease Modifying Anti-Rheumatic Drugs). On the other hand, statistically significant differences were found between biologic-naïve patients and those previously exposed to biologic drugs. Additionally, the median time of disease duration was significantly longer in patients discontinuing IL-1 blockers compared to the group retained on the treatment (5.88 vs. 3.17 years). Also, treatment delay was significantly longer in patients discontinuing treatment with IL-1 inhibitors (3.71 vs. 1.18 years), highlighting the importance of timely treatment and its impact on the long-term outcomes (Sota et al., 12-2018).

A registry-based (CARRA, Childhood Arthritis and Rheumatology Research Alliance) multicenter prospective observational pilot study by Kimura et al. (2017) attempted to evaluate different treatment approaches as the Initial Consensus Treatment Plan in 30 mostly untreated and newly diagnosed patients. The treatment strategies included GCs alone or in combination with csDMARDs, IL-6 inhibitors and IL-1 inhibitors (anakinra with potential switch to canakinumab). Overall, the use of IL-1 inhibitors led to clinically inactive disease (no active arthritis, PGA = 0, normal ESR and/or CRP, no features of systemic JIA) in 41.7% of the patients. (Kimura et al., 2017). The comparison of different treatment options was also performed by Woerner et al. (2015) who analyzed data from the CEMARA (Center des MAladies RAres) registry. Overall, clinically inactive disease (absence of systemic symptoms, active joints and morning stiffness, PGA ≤10 mm) on different biologic drugs was achieved and maintained in 48.1% of the patients without change of the biological agent. This was observed in 33/61 patients on anti-IL-1 treatment, in 2/2 patients with tocilizumab and in 1/1 patient with abatacept, but only in 1 of the 13 patients who received anti-TNF as a first-line therapy. Switching to second-line therapy was indicated in 44.2% of patients, to third-line therapy in 23.4% and to fourth-line therapy in 5.2%. The most common reasons for therapy switching were the lack of effectiveness (58.9%), loss of response (21.4%) and AEs (12.5%). The highest rates of patients with clinically inactive disease were seen in patients treated with anakinra (44.1%), canakinumab (41.9%) and tocilizumab (45%) and only in 5.9% of patients with etanercept. Treatment with TNFα inhibitors led to a particular improvement in the musculoskeletal domain (in terms of ACR Pedi 30). The response rate to anakinra was strikingly different between biological-naive and biological-experienced patients. Drug survival of anakinra as second/third biologic drug was only 43% after 12 months of treatment, compared with 63% on canakinumab and 82% on tocilizumab. (Woerner et al., 2015). The efficacy of biologic drugs in mostly biologic naïve patients was assessed in the other registry-based (BCRD, Biologics for Children with Rheumatic Diseases) study by Kearsley-Fleet et al. (2019). The patients were treated with either tocilizumab (54/76) or anakinra (22/76). At month 12, 51%, 42% and 39% of all patients on biologics achieved minimal disease activity (PGA <3.4cm, Patient Global Evaluation- PGE <2.1cm, ≤ active joint), ACR Pedi 90 response and clinically inactive disease (no active joints, no systemic features, no active uveitis, PGA = 0 and normal ESR), respectively, as the primary outcomes of the study. The differences between the tocilizumab and anakinra groups were not significant. However, a higher treatment survival rate was seen with tocilizumab (89% vs. 59%, *p* = 0.002) (Kearsley-Fleet et al., 2019).

Efficacy and safety data on canakinumab-treated patients were analyzed by Feist et al. (2018) from 4 SJIA studies (NCT00426218, NCT00886769, NCT00889863 and NCT00891046) in different age groups. A significant improvement was seen in both clinical and laboratory parameters in all groups within 15 days of treatment, and at least 50% of patients in each age group achieved a temperature decrease and adapted ACR Pedi 70 response. The favorable response to treatment lasted during the 85-days follow-up. (Feist et al., 8-2018). Afterward, Ruperto et al. (2018) published the results of the 5-years long-term extension of these trials. At the end of the study, JADAS low disease activity was achieved in 48.6% of patients (44.6% in the first 6 months). A more pronounced decrease in disease activity was found in biologic-naïve patents, while no difference was seen between patients on MTX or alone. The clinical improvement was also accompanied by a decrease in CRP and serum levels of fibrinogen. (Ruperto et al., 2018). Quartier et al. (2020) tried to resolve a question, whether dose tapering, interval prolongation or discontinuation of canakinumab may have any impact on the duration of clinical remission (no joints with active arthritis; no fever due to sJIA; no rash, serositis, splenomegaly, hepatomegaly nor generalized lymphadenopathy attributable to sJIA; normal CRP serum levels Physician Global Assessment- PGA ≤10 mm). At week 24, 71% of patients with a reduced dose to 2 mg/kg every 4 weeks and 84% of patients with a prolonged interval to 8 weeks maintained clinical remission, and further reduction of the dose or interval prolongation was feasible without disease flares in the majority of the them. However, only 33% of thepatients discontinued canakinumab and remained in remission. (Quartier et al., 2020). In a recently published trial, Brunner et al. (2020) compared the treatment efficacy of canakinumab in patients with or without fever at treatment initiation. At baseline, patients with fever were older and had higher CRP. No significant differences were found in the disease activity reduction between the fever and non-fever groups. Overall, 30.9% and 41.5% patients were able to reach clinical remission according to JADAS and ACR, respectively by month 6. Median time to JADAS and ACR clinical remission was 57 and 30 days in patients with fever, 58 and 142 days, respectively, in those without fever. The remission was sustained until study termination. Additionally, an adapted ACR Pedi 50 response by day 15 was the strongest predictor of achieving JADAS clinical remission or glucocorticoid discontinuation. The use of MTX and no corticosteroid at baseline were associated with a higher probability of achieving ACR but not JADAS clinical remission. (Brunner et al., 2020).

Despite the fact that the most robust evidence of efficacy and safety of IL-1 inhibitors exists for anakinra, and canakinumab, rilonacept also demonstrated their potential in the treatment of sJIA. The efficacy and safety profile of rilonacept were evaluated in patients with SJIA in two studies. The first multicenter, randomized, placebo-controlled study by Lovell et al. (2013). enrolled patients who had the presence of active arthritis and at least one day of systemic symptoms (fever and/or rash). At week 4, there was no significant difference in ACR Pedi 30, 50, or 70 between the placebo and rilonacept groups. However, a reduction in inflammatory parameters and clinically significant decrease in systemic manifestations (65% of rilonacept vs. 43% of placebo patients) was observed. At the 12-months time point, the most significant decrease in ACR Pedi 30, 50, and 70 was seen in 23 patients remaining in the study, and the response rates were 91.3%, 87%, and 82.6%, respectively. Two patients achieved complete remission. (Lovell et al., 2013).

A second multicenter study on the efficacy and safety of rilonacept RAPPORT was conducted by Ilowite et al. (2014) and was focused on patients with active joint involvement. In this study, the time to response (ACR Pedi 30, absence of fever, tapered GC) as primary endpoint was significantly shorter in patients treated with rilonacept in previous 4 weeks (randomized placebo control part) compared to those with placebo—4 vs. 8 weeks. Moreover, a significantly higher primary endpoint response rate (57 vs. 27%) and proportion of ACR 30/50/70 responders was observed in rilonacept group at week 4. During the whole active phase (week 4–24), the response was achieved in 77% of patients receiving active treatment and 59% of patients who had received previous placebo treatment at week 12. No influence of previous exposition to anakinra and absence of systemic manifestation on treatment response was seen. (Ilowite et al., 2014).

### Adult-Onset Still´s Disease

AOSD is a systemic inflammatory disease of unclear etiology. The disease itself is characterized by a dysregulation of the innate immune response. The clinical and laboratory manifestations involve recurrent spiking fevers, typical rash, arthralgia, sore throat, splenomegaly, lymphadenopathy, leukocytosis, and negativity of rheumatoid factors and antinuclear autoantibodies (Efthimiou et al., 2006). The disease can present in a monocyclic, polycyclic, or chronic manner with predominant joint involvement. Similarly to SJIA, the most severe complications include progressive polyarthritis leading to ankylosis (most often carpometacarpal joint), amyloidosis, and life-threatening MAS. The treatment of AOSD includes NSAIDs (Non-steroidal Anti-Inflammatory Drugs) and GCs as the first-line treatment. As a second-line treatment, MTX might be used as a disease-modifying drug contributing to the reduction or withdrawal of systemic GCs. Therefore, MTX lowers the risk of developing severe complications associated with the use of GCs. Although first- and second-line therapy have been shown to have therapeutic efficacy, clinical remission or low disease activity cannot be achieved in a large proportion of patients. These patients have AOSD with either predominant systemic features or predominant joint impairment. Thus, biological treatment is indicated in such patients who are refractory to conventional treatment. Biological therapy targets the key cytokines that are involved in the pathogenesis of AOSD (IL-1, IL-6, and IL-18) (Feist et al., 10-2018; Giacomelli et al., 2018). Anakinra was the first biological drug that was shown to be efficacious in the treatment of both the systemic and joint manifestations of AOSD. The most recent studies even support anakinra monotherapy as an effective treatment with high rates of complete responses and a significant corticosteroid sparing effect, as shown in a systemic review of literature by Giacomelli et al. GC-sparing effect was also confirmed in a recent meta-analysis by Ruscitti et al. The authors suggested that a reduction of concomitant GCs dosage following anakinra treatment is safe and does not lead to a disease flare (Ruscitti et al., 2020).

However, the efficacy has not yet been demonstrated in a randomized placebo-controlled study. The current evidence stems from several observational and open-label studies (Ruscitti et al., 2017). Other anti-IL-1 therapeutic modalities in the treatment of AOSD include canakinumab and rilonacept.

### Interleukin-1 Blockade in the Treatment of AOSD

One of the first proof-of-concept study with anakinra focused on the treatment efficacy was provided by Lequerre et al. (2008). Similarly to the SJIA patients, who were also enrolled into this study, the authors described prompt and dramatic improvement in laboratory as well as clinical manifestations in more than 70% of patients. At month 3, the complete response (improvement of ACR or ACR pedi score by 50% or more) was achieved in 60% of patients and the response persisted until the end of the study (Lequerre et al., 2008). Even higher response rate was observed in a case series study by Laskari et al. (2011). Complete response (resolution of all disease-related symptoms, except of joint erosion) was seen in 80% of patients at the end of the study. On the other hand, the treatment had no effect on proteinuria in a patient with suspected amyloidosis. (Laskari et al., 2011). These findings were also confirmed in other retrospective studies (Giampietro et al., 2013; Iliou et al., 2013) However, Ortiz-Sanjuán et al. (2015) observed only a limited potency of anakinra to improve the articular symptoms. Whereas the frequency of cutaneous manifestation was reduced after 1 year of therapy from 58.5% at baseline to 7.5%, fever from 78 to 14.6%, and lymphadenopathy from 26.8 to 4.9%, the frequency of joint manifestation was reduced from 87.8 to 41.5% only. A higher response rate was observed in patients on concomitant medication with MTX. (Ortiz-Sanjuan et al., 2015). A limited response in the musculoskeletal domain was also shown by Cavalli et al. (2015) in a retrospective study. In this study, biologics were indicated upon GCs and/or csDMARDs failure. Anakinra led to a complete response (normalization of CRP and ESR, reduction of the GC use of at least 50% for at least 2 months) in systemic manifestation in 92% of patients, but only in 37% of patients with chronic articular symptoms. 25% of patients with chronic articular symptoms reached a partial remission, and 25% of patients failed to respond. On the other hand, these patients responded to tocilizumab as a second-line therapy. Additionally, the authors also concluded that TNFα blockers do not represent a valuable treatment options (Cavalli et al., 2015). Insufficient efficacy of TNFα blockers was later confirmed in a retrospective study including a large cohort by Sfriso et al. (2016) **.** In this study, complete, partial or no response to anakinra therapy was seen in 74, 20, and 2% of patients, respectively, compared to response rates of TNFα inhibitors—22% (complete), 24% (partial), and 54% (no response) (Sfriso et al., 2016). Interestingly, Dall´Ara et al. (2015) identified the presence of pericarditis as a significant predictor of bDMARd need (OR = 3.62, 95% CI = 1.22 to 10.7, *p* = 0.028). Other assessed clinical and laboratory features, including fever, sore throat, skin rash, arthritis, lymphadenopathy, splenomegaly, leukocytosis, and increased liver enzymes, did not reach significant differences. These findings open up the question of therapeutic use and efficacy of anakinra and other IL-1 inhibitors in other conditions, such as recurrent idiopathic pericarditis, where a role of the IL-1 cytokine family has also been broadly discussed (Dall'Ara et al., 2016).

One of the first randomized studies that focused on the use of anakinra in refractory AOSD patients was performed by Nordström et al. (2012) **.** The authors demonstrated the beneficial effect of IL-1 inhibition with anakinra in patients with refractory AOSD in comparison to csDMARDs. The efficacy was assessed at weeks 4, 8 and 24. Complete remission (normal body temperature, absence of NSAIDs use, CRP and ferritin within reference limits, no swollen and tender joint) was achieved in 50% of patients with anakinra vs. 30% of patients with csDMARDs at week 4, 58 vs. 50% at week 8 and 50% vs. 20% at week 24. CRP normalized without significant differences in both groups at week 8. (Nordstrom et al., 2012).

These findings should be verified by randomized, double-blind, placebo-controlled phase III, which is currently ongoing (NCT03265132). Preliminary results seem to be very promising. The study was completed by all included patients, and neither AEs nor lack of efficacy were reported (Clinical Trails, 2020). Although the results of the study are not yet available, the administration of anakinra to AODS patients was approved by the EMA (European Medicines Agency, 2019a) in 2018 (accessed on March 8, 2019).

The use of anakinra was also associated with favourable DRR. Sota et al. (2019) in a cohort consisted of AOSD and SJIA patients revealed cumulative DDR that varied from 74.3% at month 12–49.4% at month 48 and persisted until the end of study at month 60 without any significant differences between AOSD and SJIA patients or between patients on combined therapy with csDMSRDs and monotherapy. On the other hand DDR was significantly lower in patients previously treated with other biologics compared to biologic-naïve patients and was negatively influenced by the treatment delay (4 years in patients with treatment discontinuation vs. 0.66 years in those that retained it) and occurrence of AEs (Sota et al., 2019).

DRR was also reported in a recent study by Vitale et al. (2019) assessing the efficacy of anakinra in association with different factors. While it has been shown that anakinra has an excellent DRR: 44.6% and 30.5% at month 60, 120 respectively, the risk of losing the therapeutic efficacy was associated with the number of swollen joints at the start of therapy. The percentage of patients retained on therapy further increased after exclusion of AEs and long-term remission as the reasons for discontinuation to 68.2% (month 60) and 54.6% (month 120). In contrast, in the report by Sota et al. (2019), the overall DRR did not differ in biologic naïve patients and those previously treated with other biologics. Similar observations were found in patients treated with csDMARDs. Moreover, the type of AODS (systemic versus chronic articular), the disease duration or the age at the disease onset did not define the response to anakinra treatment (Vitale et al., 2019).

Regarding the concept of “window of opportunity” in SJIA, Vitale et al. (2020) also attempted to resolve the question regarding the significance of early therapeutic intervention in affecting the disease course. The study was conducted in the same cohort as the previous study. The patients initiating anakinra therapy within 6 and 12 months since the disease onset showed significantly faster reduction of CRP and ESR compared to the patients with later treatment initiation. There was also faster to decrease in the number of affected joints within 3 months of therapy in patients starting anakinra before month 6. The treatment initiation within first 12 months since the AOSD onset was associated with a significantly faster decrease of ESR and CRP. Nevertheless, the differences were lost at month 6 and 12. To conclude, clinical and therapeutic outcomes are independent of early anakinra treatment initiation. However, faster response to control systemic and articular manifestation may be observed in patients with early intervention after disease onset (Vitale et al., 2-2020).

Canakinumab represents another potential IL-1 inhibitor indicated in AOSD patients that may be considered a treatment of choice in cases where anakinra therapy has failed or has not led to a sufficient clinical response. Before the CONSIDER study by Kedor et al. (2020) was published, only limited evidence for the efficacy of canakinumab in patients with AOSD was available in the literature, partly in the form of single case reports or case series (Kontzias and Efthimiou, 2012; Banse et al., 2013; Barsotti et al., 2014; Lo Gullo et al., 2014; Rossi-Semerano et al., 2015). 7). The primary endpoint was focused on articular manifestation and defined by a change in disease activity score ΔDAS28 (ESR) > 1.2 points. This endpoint was achieved in 66.7% of the patients treated with canakinumab and in 41.2% of patients treated with placebo at week 12. However, this difference was not statistically significant, and the anticipated outcome was therefore not met. Despite these results, the EMA approved the use of canakinumab in patients with AOSD (Kedor et al., 2020). Notably, gene expression profiles in AOSD and SJIA, as well as other disease aspects, display overlapping and nearly identical clinical patterns (Nirmala et al., 2015). The administration of canakinumab in patients with AOSD is, thus, a rational approach, and a positive effect on the disease outcome is expected.

In contrast to the results of previous study, clinical efficacy of canakinumab in real-life practice was confirmed in a retrospective study by Vitale et al. (2020). All enrolled patients experienced disease relapse before canakinumab initiation. Complete control was achieved in almost 90% of patients within 3 months and persisted until the last assessment at month 9, 12, respectively. Improvement occurred in all aspects of the disease–systemic (systemic severity score) and musculoskeletal manifestation (TJC, SJC and DAS28-CRP), as well as laboratory markers of inflammation (leukocytosis, serum level of ferritin, CRP and ESR) that returned to normal values. No differences were found between patients treated with monotherapy compared to patients treated with a combination therapy with csDMARDs in the disease duration and the system severity score at baseline. In this study, arthritis was more frequent among patients requiring combination therapy (Vitale et al., 10-2020). However, clinical studies are still needed.

Rilonacept is the most novel IL-1 inhibitor available. Several clinical trials with AODS patients are expected to announce the results. In a systematic review of 11 AODS cases, rilonacept was used in patients refractory to NSAIDs, DMARDs, and even anakinra. In 65% of the patients, complete remission of the disease was achieved. Partial remission was seen in the remaining 45%. The individual studies differed significantly in dosing regimens. However, the usual dose ranged from 160 mg to 320 mg given once a week or as needed, therapy for joint symptoms required higher doses (up to 360 mg) (Junge et al., 2017).

The inhibition of another member of IL-1 family, IL-18 may present next promising therapeutic approach in patients with AOSD. Currently, only the data from the open-label study by Gabay et al. (2018) are available. The study focused on the safety and efficacy of the treatment. Patients were sequentially treated with either 80 mg or 160 mg tadekinig alfa three times per week for 12 weeks. Overall, tadekinig alfa was well tolerated, and adverse reactions were predominantly local reactions at the injection site, followed by infections, with a total of 3 SAEs reported during the study duration. The predefined response criteria (included reduction in TSC44 and SJC44, decrease of CRP or ferritin serum level) were achieved in 44.4–58.3% of patients. However, more clinical trials are needed to confirm these results (Gabay et al., 2018).

### Idiopathic Recurrent Pericarditis

IRP is an autoinflammatory disorder with recurrent episodes of sterile inflammation that occurs as a relatively common complication (15–30%) of acute pericarditis (Lazaros et al., 2017). The IRP patients experience episodes of disease recurrence usually within 3–6 weeks. Therefore, the disease becomes chronic and may be associated with significant morbidity affecting the patient’s quality of life. (Imazio et al., 2016). Even though, the long-term prognosis of IRP is relatively good and associated with a low risk of developing constrictive pericarditis, the current treatment options may not be sufficient to prevent the disease recurrence. The mainstay therapy preventing the acute pericarditis recurrence is the administration of NSAIDs and colchicine. The use of corticosteroids is also widespread and especially in children, related to a threatening risk of development of GC-dependent disease with associated side effects. A great hurdle in the treatment of IRP remains the colchicine resistance which occurs in some IRP patients (Tombetti et al., 2020). The mechanisms underlying the colchicine resistance have not yet been clarified, however, the inadequate responses to colchicine have been also observed throughout various autoinflammatory disorders, such as Familial Mediterranean Fever (Ozen et al., 2017; El Hasbani et al., 2019).

Because IRP is accompanied by an increased IL-1 production, the suppression of IL-1 can affect the disease activity and significantly improve the clinical manifestation of IRP patients, especially of those with colchicine resistance (Scott et al., 2011; Cantarini et al., 2015; Lazaros et al., 2017).

### IL-1 Blockade in the Treatment of IRP

Several observational studies summarized data on the efficacy of anakinra in pediatric (Picco et al., 2009; Scardapane et al., 2013; Finetti et al., 2014; Murias Loza et al., 2018) and adult IRP patients (Vassilopoulos et al., 2012; Lazaros et al., 2014). The results of a single randomized AIRTRIP study by Brucato et al. (2016) involving 34 patients with IRP were impressive. All patients experienced a rapid and sustained response to treatment. During a follow-up period, pericarditis recurred in 18% of patients in the anakinra cohort, in contrast to the placebo cohort, in which 90% of patients experienced pericarditis recurrence. (Brucato et al., 2016).

Anakinra was also shown to be a promising and safe treatment for colchicine-resistant, GC-dependent or in case of intolerance of other therapeutic modalities in a large cohort of 110 patients shown by Imazio et al. (2016). The authors showed a significant (*p* < 0.05) drop in the frequency of recurrence from 4.29/year before study initiation to 0.14/year in patients who failed in response to the standard therapy (Imazio et al., 2016).

These finding were confirmed later by Imazio et al. (2020) in a registry based study IRAP (International Registry for Anakinra in Pericarditis) with more than 200 patients with colchicine-resistant and GC- dependent recurrent pericarditis. Anakinra therapy led to significant reduction of flares during follow-up period of 36 months by 83% from incidence 2.33 to 0.39 flares per patient/year. Only 28% patients had more than 1 flare at month 36. The flare-free intervals were also prolonged from the mean 157 days to 10 months (Imazio et al., 2020). However, preventing pericarditis recurrence after anakinra withdrawal remains a challenge (Picco et al., 2009; Scardapane et al., 2013; Finetti et al., 2014; Brucato et al., 2016; Murias Loza et al., 2018).

The efficacy of canakinumab in IRP has not yet been evaluated. To date, only a few case reports or case series have shown the efficacy and safety of canakinumab in pediatric as well as adult IRP patients. The AEs or ineffectiveness of previous treatments were the most common reasons for canakinumab administration (Kougkas et al., 2018; Epcacan et al., 2019). Similar to the unresolved question of canakinumab efficacy in patients with IRP, the results of a pilot open-label study with rilonacept (finished in 2019) are expected (NCT03980522). A randomized double-blind placebo-controlled study RHAPSODY (NCT03737110) should be completed in June 2020 (Clinical Trial, 2020).

### Kawasaki Disease

KD is an acute inflammatory vasculitis of unknown etiology that is usually observed in childhood. Untreated KD is associated with a significantly higher risk of coronary artery abnormalities, thromboembolic occlusions, and myocardial infarction, with a consequent increased risk of death (Marrani et al., 2018). The etiology of KD remains unclear, although different theories regarding the complex pathogenesis of Kawasaki disease have been proposed (Principi et al., 2013). Currently, there is some evidence that KD is associated with infection. Intravenous immunoglobulins (IVIG), in combination with aspirin, are the recommended first-line treatment for KD. The administration of IVIG during the first ten days after the onset of fever decreases the risk of developing coronary artery aneurysms five-fold (De Rosa et al., 2007). The biologic nature of KD and a younger age at disease onset may affect the patient's response to IVIG therapy (Rigante et al., 2016). Approximately 10–15% of the patients do not respond to IVIG therapy. The prediction of IVIG nonresponders is crucial. Early identification of high-risk patients may allow more intensive treatment. Moreover, in patients receiving intensive treatment and IVIG therapy, successful prevention of coronary artery disease may be achieved (Agarwal and Agrawal 2017). To date, there is only limited evidence of a therapeutic effect of IL-1 inhibition in children with KD (Cohen et al., 2012; Sanchez-Manubens et al., 2014; Shafferman et al., 2014; Agarwal and Agrawal 2017; Blonz et al., 2018; Guillaume et al., 2018).

### IL-1 Blockade in the Treatment of KD

A retrospective case series study by Koné-Paut et al. (2018) evaluated the administration of anakinra in children with who were refractory to standard treatment and had signs of persistent inflammation, a progression of coronary dilation, and severe myocarditis with heart failure. Anakinra has been shown to be effective at controlling KD, with fever and inflammatory parameters disappearing in all patients. In addition, coronary artery dilatation improved in more than 90% of patients. (Kone-Paut et al., 2018).

Recently, the results of the KAWAKINRA study by Kone-Paut et al. (2020)—proof-of-concept study (open label) in 16 patients refractory to intravenous immunoglobulins were published. The administration led to decreased temperature (primary endpoint) in 75% of patients as well as improvement in the secondary outcomes: decreased PGA score, reduction of CRP levels and z-score reflecting damage of coronary arteries. All SAEs required prolonged hospitalization, but they resolved upon cessation of anakinra (Kone-Paut et al., 2020). Another open-label prospective study (NCT02179853) initiated in November 2014 is currently ongoing, and study completion is expected in December 2020 (Clinical Trials, 2020).

### Behçet's Syndrome

BS represents a systemic vasculitis of unknown etiology affecting mostly small and large vessels of arterial as well as venous system. BS is currently considered a disease with overlapping manifestations of autoimmune and autoinflammatory syndromes and exhibits different clusters of symptoms including recurrent oral aphthae, skin lesions, arthritis, uveitis, thrombophlebitis and gastrointestinal, as well as neurological manifestation (Yazici et al., 2010). The unspecific clinical manifestation, histology and laboratory findings make the diagnostic process challenging (Yazici and Yazici 2014). Despite of these limitations, diagnostic criteria were introduced by Hatemi et al., 2008 (Hatemi et al., 2008) and updated by Davatchi et al**.** in 2013 (International Team for the Revision of the International Criteria for Behcet's Disease, 2014). New treatment strategies have been studied over the last several years. Because IL-1 has been shown to represent a key proinflammatory cytokine in the BS pathogenesis, the IL-1 blockade may be a reasonable approach for the disease treatment apart from TNFα inhibitors (Fabiani et al., 2017; Yazici, 2020)which are according to EULAR (European League Against Rheumatism) recommendations for BS management currently used for the treatment of the majority of BS complications (Hatemi et al., 2018) The most favorable treatment results were observed particularly in association to anakinra and canakinumab treatment and showed efficacy in controlling of ocular manifestation. On the other hand, evidence of anakinra/canakinumab capability to affect mucocutaneous and musculoskeletal manifestation, together with the prevention of secondary amyloidosis, is limited. The use of gevokizumab in BS patients remains controversial (Bettiol et al., 2019).

### IL-1 Blockade in the Treatment of Behçet's Syndrome

First experience with use of IL-1 inhibitors in BS comes from single case reports and case series (Botsios et al., 2008; Cantarini et al., 2012; Ugurlu et al., 2012; Vitale et al., 2014) A pilot study focused on the efficacy and safety of anakinra in 6 BS patients were performed by Grayson et al. (2017). The therapy led to 2 complete (no evidence of organ-threatening disease and ocular manifestation) and 2 partial responses (decrease in number of genital or oral ulcers from baseline) (Grayson et al., 2017).

Emmi et al. (2016) confirmed a favorable safety profile and good adherence to the treatment in a multicenter retrospective study. Median time on therapy was 6 and 8 months in patients treated with anakinra and canakinumab, respectively, as an initial therapy. The medians were even higher with therapy adjustments – 10 and 13 months along with high overall cumulative survival up to 71.6% at month 23 in canakinumab group. Better response was seen in higher doses of anakinra. Moreover, canakinumab showed efficacy after failure of a first line IL-1 inhibitor (Emmi et al., 2016).

Disease duration and ocular manifestation were found to be potential predictors of complete response to IL-1 inhibitors in a retrospective study by Fabiani et al. (2020). The patients treated with both anakinra and canakinumab were divided into 2 groups–complete responders defined as patients with sustained response for 52 weeks at least (group 1) and non-responders with primary or secondary ineffectivity (group 2). In group 1, complete response was achieved within 3 months after therapy initiation in all patients, disease relapse occurred in median time of 79.7 weeks. The efficacy was recovered by adding of MTX to anakinra or by shortening of canakinumab administration intervals up to every 4 weeks. In comparison to 38.9% of complete responders and 18 weeks long relapse-free interval in group 2. Both groups did not differ neither in demographics nor disease activity, manifestation and treatment including previous treatment option or used IL-1 inhibitor with the exception of the frequency of ocular manifestation (66.7% in group 1 vs. 16.7% in group 2) and disease duration (15.8 years vs. 8.7 years) (Fabiani et al., 2020).

Despite of the promising results of open-label pilot study (Gül et al., 2012) (Gul et al., 2012), the multicenter prospective randomized placebo controlled trial by Tugal-Tutkun et al. (2018) did not meet the primary endpoint to demonstrate superiority of gevokizumab over placebo in reduction of the risk of BS-related uveitis exacerbations (Tugal-Tutkun et al., 2018).

### Crystal-Induced Arthropathies

Inflammatory crystal-induced arthropathies are heterogenous group of rheumatologic diseases with an overlapping symptomatology. This group typically comprises gout and CPDD (also called pseudogout). Despite the fact that both are regarded as polygenic and multifactorial diseases, monogenic variants and susceptibility gene loci have been identified (Dalbeth et al., 2017; Williams et al., 2018). While the gout is driven by prolonged hyperuricaemia forming natrium urate crystals that accumulate in joints and other tissues, CPDD is characterized by an overproduction of extracellular inorganic pyrophosphate by chondrocytes. Tissue deposits of both can activate the components of NLRP3 inflammasome leading to caspase-1 mediated activation of IL-1β (Figure 1). IL-1β further promotes inflammatory response that is characterized by spontaneous attacks of pain and swelling. Based on the revealed pathogenesis of gout and CPDD, there is a rationale for the use of IL-1 inhibitors for the therapy (Dalbeth et al., 2016; Rosenthal and Ryan 2016). The efficacy of IL-1 inhibitors in the treatment of crystal-induced arthropathies has been successfully tested in clinical trials (Macmullan and McCarthy 2012; Kluck et al., 10-2020). Based on these results selected inhibitors have become a part of the recommendations for the treatment of gout in a patient with unresponsiveness to standard therapy or contraindications (Richette et al., 2017).

### IL-1 Blockade in the Treatment of Gout and Calcium Pyrophosphate Deposition Disease

A pilot proof-of-concept study with anakinra designed as case series of 10 patients who did not respond to standard therapy including NSAIDs, GCs and colchicine was performed by So et al. (2007). All patients responded within 48 h. Moderate response was seen in patients with tophaceous gout. No treatment-related side effects were observed during therapy and there were no infectious complications (So et al., 2007). Favorable efficacy and safety profile was also described in the other retrospective observations (Chen et al., 2010; Ghosh et al., 2013)**.** Apart from efficacy and safety, Ottaviani et al. (2013) retrospectively analyzed the effect of anakinra and the risk of relapses in a cohort of 40 patients with acute gout attack. Similarly to previous reports, good response was seen in 90% of patients. The therapy led to significant reduction of pain score (from 73.5/100 mm at baseline to 25/100 mm) and CRP (from 130.5 mg/L to 16.0 mg/L). Relapses were reported in 32.5% of patients in follow-up period of 7 months. No relapse occurred in patients receiving anakinra as a long-term therapy (>15 days) (Ottaviani et al. 2013). So far, a single randomized, double-blind, placebo - controlled study by Janssen et al. (2018) was conducted. The study showed non-inferiority of anakinra treatment in reduction of pain (primary endpoint). Moreover both therapeutic approaches led to improvement in PGE and decrease of CRP (Janssen et al., 2019). The results of other study - anaGO, a randomized, double-blind, controlled trial (NCT03002974) assessing the efficacy of anakinra compared to intramuscular triamcinolone and placebo in acute gout flare, are expected. The study was completed in August 2019 (Clinical Trials, 2020), Another treatment option for the patients with insufficient response to standard therapy represents canakinumab. A randomized single-blind, active controlled trial by So et al. (2010) with different dosing regimen ranging from 10 mg to 150 mg s. c. in a cohort of 200 patients with acute gouty attack showed significant reduction in pain intensity (primary endpoint) compared to triamcinolone 40 mg intraarticularly. The analysis at 40 h time point after therapy initiation revealed equivalent efficacy of canakinumab 23 mg to triamcinolone and at 72 h even all doses of canakinumab were associated with better response. The response to canakinumab positively correlated with the increasing dose. Canakinumab 150 mg led to up to 18.2 mm greater decrease on 100 mm–VAS compared to triamcinolone. There was also significantly longer interval to relapse in the canakinumab group. At week 8, only 1 patient in the canakinumab group experienced flare compared to 25 patients treated with triamcinolone. Significantly greater impact of canakinumab was also observed in all investigated secondary endpoints–PGA, PGE, CRP and SAA (So et al. 2010).

A randomized, double-blind, active-controlled trial followed by double-blind extension by Schleisinger et al. (2012) investigated the efficacy of canakinumab in core study (β-RELIEVED) and in the study extension (β-RELIEVED II). The administration of canakinumab led to a significant reduction of pain from 73.3 mm on 100 mm-VAS at baseline to 25.0 mm that was superior to triamcinolone (reduction from 74.8/100mm to 35.7/100 mm), thus, the study met the primary endpoint. Fewer patients also required the use of rescue medication (37.3 vs. 54.6%). Positive responses were also achieved in other secondary objectives. The effect of the therapy was further sustained during the extension period. At week 24, the overall incidence of flares was reduced by 56% in the canakinumab group compared to the triamcinolone group (0.4 event per patient vs. 0.87). The median time to first attack was also significantly prolonged (>186 days vs. 131 days) (Schlesinger et al. 2012).

Canakinumab was also tested in the prevention of gouty flares during uric acid-lowering therapy in a randomized, double-blind, active-controlled trial by Schlesinger et al. (2011). Canakinumab was assessed in 6 different dosing regimens in comparison to colchicine. Canakinumab 200 mg and 300 mg led to a significantly lower frequency of flares than colchicine. The doses ≥50 mg of canakinumab reduced the number of attacks by 62–72% in comparison to colchicine (rate ratio 0.18–0.38). The proportion of patients who experienced ≥1 attack(s) of gout was 15–27% for all canakinumab groups vs. 44% in colchicine treated patients. Prevention of flares with canakinumab was also associated with longer interval to the first attack and shorter duration of flares. In all canakinumab groups CRP was consistently lower than in colchicine and were below the upper limit of the range (≤3 mg/dl) until week 24 (Schlesinger et al. 2011).

The efficacy of IL-1 inhibition during acute flares of gout was also expected in rilonacept. Proof–of–concept trial by Terkeltaub et al. (2009) primary focused primarily on safety of rilonacept also evaluated its efficacy. Overall, AEs were reported as rare, particularly ISRs were observed. No death or SAE were reported. Compared to placebo, rilonacept treated patients achieved significant reduction of pain on 10-point VAS from 5.0 at baseline to 2.8 after 2 weeks of active treatment. The response sustained until week 6 accompanied by improvement in PGA as well as PGE. The therapy with rilonacept also led to significant reduction in hs-CRP (Terkeltaub et al., 2009).

Based on the favourable results of proof-of-concept study the efficacy of rilonacept was also assessed in a randomized, double-blind, active- and placebo-controlled study by Terkeltaub et al. (2013). The efficacy of rilonacept monotherapy, rilonacept with indomethacin and monotherapy of indomethacin was compared. All treatments led to a significant reduction in pain from baseline at 24, 48 and 72 h. However, the mean differences of pain reduction between rilonacept monotherapy and rilonacept plus indomethacin group were not statistically significant (1.55 vs. 1.40 point on Liskert scale). Therefore, the primary endpoint of the study was not met. Moreover, separate ad hoc analysis showed favorable response of indomethacin monotherapy over rilonacept alone. Similar results were obtained when 11-point rating scale was used. There was also similar proportion of patients requiring rescue therapy in both groups. On the other hand, rilonacept had greater impact on hsCRP reduction (Terkeltaub et al., 2013).

In contrast to the previous study, rilonacept has shown to serve as a very effective prevention of acute gouty flares during uric-acid lowering therapy in a randomized, double-blind, placebo-controlled by Sundy et al. (2014). Mean serum levels of uric acid at baseline (8.2 and 8.0 8.0 mg/dl), at endpoint (5.8 and 5.7 mg/dle) and dose of allopurinol (342.4 and 325.4 mg/day) were without significant differences between placebo and rilonacept groups. The rilonacept group at week 16 displayed fewer gout flares per patient (1.73 vs. 0.51 flare per patient). The proportion of patients with ≥1 flare(s) was 51.1% in placebo and 25.7% in rilonacept group. There was also significantly shorter duration of particular attacks in the rilonacept group–mean 2.7 days vs. 7.7 days in the placebo group. More attacks were experienced by patients with tophus gout (Sundy et al., 2014). Similar results were achieved also in previously conducted phase III trials (Schumacher et al., 2012; Mitha et al., 2013).

There is also an ongoing open-label randomized active-controlled phase II trial (NCT04067492) with novel IL-1 inhibitor RPH 104 (heterodimeric fusion protein compound of human extracellular portions of IL-1RI and IL-1 receptor accessory protein, each linked to a mutant Fc portion of human IgG1), that compares its efficacy and safety to diclofenac in patients with gout attack (Clinical Trials, 2020). RPH 104 is also tested in Schnitzler Syndrome (NCT04213274) (Clinical Trials, 2020). Other IL-1 inhibitors such as Dapansutrile (an oral selective inhibitor of NLRP3 inflammasome) with different mechanism of action have been also successfully assessed as a potential therapeutic approach in patients with acute gouty flares (Kluck et al., 10-2020).

In contrast to gout, there is very limited evidence of the use of IL-1 inhibition in CPDD patients. This evidence comes primarily from individual case reports or case series. IL-1 inhibition was indicated particularly in cases of unresponsiveness to standard therapy or contraindications. Anakinra was used as the preferable IL-1 inhibitor. Nevertheless, randomized controlled and long-term trials are further required (Rosenthal and Ryan, 2016; Iqbal et al., 2019).

### IL-1 Blockade in the Other Inflammatory Diseases

There is a broad spectrum of other diseases sharing autoinflammatory signatures. In such diseases, the efficacy of IL-1 inhibitors may be expected. Schnitzler syndrome characterized by chronic urticarial, fever, and development of hematopoietic malignancies, as well as Waldenström macroglobulinemia, and SAPHO syndrome, might be affected by IL-1 blockade. SAPHO syndrome is a chronic immune-mediated condition affecting skin, joints, and bones. The syndrome’s acronym reflects the main features - synovitis, acne, pustulosis, hyperostosis, and osteitis. Schnitzler syndrome represents a rare disease with a great potential for the use of IL-1 inhibition which is described in patients with anakinra treatment, canakinumab, and also with the rilonacept where the therapeutical efficacy was observed in up to 94% of the cases. In Schnitzler syndrome, IL-1 inhibitors are suggested and recommended as a first-line therapy despite the lack of randomized controlled trials (Krause et al., 2012; Krause et al., 2017). The positive effect of IL-1 inhibition, was also described in patients with SAPHO syndrome. Anakinra as a preferable treatment option was examined upon failure of TNFα inhibitors as a first-line therapy (Firinu et al., 2016). On the other hand, the progress in understanding of pathogenesis of many seemingly unrelated conditions revealed the potential of IL-1 inhibition in other conditions, such as atherosclerosis and acute myocardial infarction, heart failure, myocarditis and dilated cardiomyopathy (Szekely and Arbel, 2018), but also type 2 diabetes and many other conditions (Cavalli and Dinarello, 2018; Gram, 2020). Here, the CANTOS study represents one of the most extensive evidence for the use of IL-1 inhibition out of range of “typical” polygenic diseases with more than 10.000 enrolled patients. The study showed a 15% reduction of nonfatal myocardial infarction (MI), nonfatal stroke, or cardiovascular death in patients who experienced myocardial infarction along with elevated CRP and were treated with canakinumab 150 mg. Nearly all of that reduction came in nonfatal MI. On the other hand, there was no significant difference in stroke, cardiovascular death, and overall mortality. Moreover, the treatment was associated with a higher incidence of fatal infection (Ridker et al., 2017).

### Safety Considerations of IL-1 Blockade

Generally, IL-1 inhibition showed favourable safety profile throughout the studies including different conditions and therapeutic regimens. The overall incidence of AEs ranged widely from 5 to 88% in large retrospective and prospective interventional trials ( So et al., 2010; Schlesinger et al., 2011; Schlesinger et al., 2012; Ottaviani et al., 2013; Terkeltaub et al., 2013; Ilowite et al., 2014; Emmi et al., 2016; Ridker et al., 2017; Feist et al., 8-2018; Gabay et al., 2018; Ruperto et al., 2018; Janssen et al., 2019; Sota et al., 2019; Fabiani et al., 2020; Imazio et al., 2020; Kone-Paut et al., 2020). The most dominant AEs were ISRs particularly reported in patients treated with anakinra and rilonacept. However, anakinra showed a favourable safety profile in critically ill and hospitalized patients endangered by infections. Infections were the second most common AEs and occurred more commonly in the canakinumab groups (Thueringer et al., 2015; Liew and Gardner, 2019)**.** Other less frequently observed AEs were complications affecting the gastrointestinal and musculoskeletal system, or hematological and liver enzymes abnormalities. The majority of AEs were reported as mild or moderate and occurred within first months of the therapy. SAEs were mostly related to the activity of underlying diseases or infections. Both AEs and SAEs significantly contributed to lower DRR. Infectious complications were observed especially in patients with co-medication of IL-1 inhibitors and csDMARDs or NSAIDs. These findings were consistent with the comprehensive retrospective study by Sota et al. (2018), who analyzed the records from 475 patients (280 females, 195 males, mean age 36.36 years) with broad spectrum of polygenic AIDs (AOSD, SJIA, BS, IRP), monogenic AIDs (FMF, CAPS, TRAPS) and other diseases, where IL-1 inhibition was indicated. Totally, 89 AEs were recorded during the mean follow up 24.39 years, 14,61% of them were reported as SAEs. ISRs and non-localized rashes were the most common. SAEs included 4 cases of anaphylaxis and 3 cases of severe bacterial infections, 2 of them led to death. Other remaining causes of death were MAS, myocarditis and severe neutropenia. The majority of AEs occurred within first 12 months after treatment initiation (51 vs. 38 events) and higher incidence was observed in older patients (≥65 years of age) and in patients with AOSD. No significant differences were found between IL-1 inhibitors in monotherapy and combination with csDMARDs. Furthermore, differences were not observed between biologic-naïve patients and those previously treated with biologic drugs. Overall, AEs occurred in 18.8% of patients within the study duration and were also one of the major reason for the discontinuation of the therapy in 8.2% patients (Sota et al. 8-2018).

### IL-1 Blockade and Combination Therapy

Important phenomenon accompanying the majority of studies was GC- sparing effect of IL-1 inhibition as a crucial point in a prevention of treatment-related complications associated with the use of GCs (Poetker and Reh, 2010). In large studies (Nordstrom et al., 2012; Ilowite et al., 2014; Junge et al., 2017; Ruperto et al., 2018; Sota et al., 2019; Vastert et al., 2019), IL-1 inhibition enabled the termination of GCs use in around 30–40% of patients without negative impact on efficacy. Higher rate of successful GCs tapering was observed particularly in patients with early initiation of anti- IL-1 therapy. GC-sparing effect of IL-1 inhibitors was significantly higher compared to csDMRDs. Moreover, comedication with csDMARDs or NSAIDs was also suspended in a significant number of patients. In contrast to TNFα inhibitors, IL-1 blockers were, therefore, effective also in monotherapy (Kalden and Schulze-Koops, 2017). On the other hand, csDMARDs such as MTX were required in patients with articular manifestation.

## Conclusion

To date, there is strong evidence that IL-1 cytokines are involved in the complex pathogenesis of the broad and continuously growing spectrum of polygenic AIDs, such as SJIA, AOSD, KD, IRP, and BS. IL-1 blockade has been proven in clinical trials to cause rapid reductions in clinical symptoms and normalization of laboratory parameters in the majority of cases. Moreover, the hematologic and biochemical parameters impressively normalized within hours to days after the first injection. In contrast to the nonspecific nature of GCs, anti-IL-1 agents present a selective treatment option for conditions with IL-1-mediated innate immunity dysregulation. The therapeutic strategies to reduce IL-1 activity provide a desired control of local and systemic inflammation and, in polygenic AIDs, improve particularly systemic symptoms. However, the efficacy in controlling the musculoskeletal manifestations is less pronounced. Most of the patients with articular involvement required concomitant therapy with csDMARDs, such as MTX. Release of articular symptoms was also seen in the TNFα inhibitors, which were not, however, effective enough to reduce systemic manifestation. This represented a significant limitation of TNFα inhibitors in the treatment of polygenic AIDs, such as SJIA or AOSD. On the other hand, these agents represent a valuable therapeutic option in other polygenic diseases, such as BS or SAPHO syndrome.

An important benefit of IL-blockade is the possibility of GCs tapering and, in many cases, even withdrawal. On the other hand, early disease flares were also reported to occur upon therapy termination even in patients with clinically inactive disease; therefore, tapering or withdrawal of treatment remains challenging. Early intervention after the disease onset seems to be another key factor of successful treatment supporting the concept of “window of opportunity.”

Therefore, the disease pathophysiology may be altered by early therapeutic intervention and the structural damages may be avoided. Currently, the verification of this concept appears to be crucial, especially in pediatric rheumatology (Nigrovic, 2014).

IL-1 inhibitors that are currently tested in clinical trials for SJIA, AOSD, KD, IRP, BS or Crystal-induced Arthropathies, such as gout or CPDD, include anakinra, rilonacept, and canakinumab. Anakinra, an IL-1 receptor antagonist, canakinumab, an anti-IL-1β monoclonal antibody, and rilonacept, a soluble decoy receptor, represent efficient therapeutic approaches to control polygenic AIDs. However, although rilonacept has been withdrawn from use in the European Union, it is currently available for the treatment of cryopyrin-associated periodic syndrome in the USA.

Due to the pharmacodynamic and pharmacokinetic properties, anakinra requires daily s. c. injections in clinical trials for the most indications. Rilonacept is administered as a single weekly injection, and canakinumab injections are given once every 4–8 weeks. The weekly administration of canakinumab favors the patients’ compliance.

Therapy with IL-1 inhibitors fulfilled the safety requirements and was generally well tolerated throughout the studies. The reported AEs were mostly ISRs (particularly with the use of anakinra) and infections, which were mild to moderate. Only limited numbers of AEs were assessed as serious and led to treatment termination or death.

Thus, all the evidence currently available strongly supports the importance of IL-1 blockade as a safe and effective therapeutic option in patients with various polygenic AIDs. The efficacy of promising anti-IL-1 therapies, such as gevokizumab, tadekinig alfa, tranilast or dapansutrile, in the treatment of polygenic AIDs remains to be clarified. The progress in the understanding of the pathogenesis of other conditions allows the use of IL-1 inhibitors beyond the conventional concept of AIDs. IL-1 dysregulation and potential use of IL-1 blockade is currently under consideration in many other diseases ranging from atherosclerosis, acute myocardial infarction, heart failure, myocarditis and dilated cardiomyopathy to type 2 diabetes mellitus and many other conditions. Interestingly, the potential use of IL-1 inhibition is in contrast to the results of TNFα inhibitors in the treatment of heart failure. TNFα blockade not only failed, but also led to the disease worsening in cases of heart failure (Kotyla, 2018).

Despite so far promising results, the ability to treat inflammatory conditions, such as polygenic AIDs, with IL-1 blockade still need more profound evaluation. Further analyses considering the reasonable costs and adverse effects of single anti-IL-1 agents are required.
